# Impact of Forward Osmosis Operating Pressure on Deformation, Efficiency and Concentration Polarisation with Novel Links to CFD

**DOI:** 10.3390/membranes11030161

**Published:** 2021-02-26

**Authors:** Alexander J. Charlton, Gaetan Blandin, Greg Leslie, Pierre Le-Clech

**Affiliations:** 1 UNESCO Centre for Membrane Science and Technology, School of Chemical Engineering, University of New South Wales (UNSW), Sydney, NSW 2052, Australia; alexander.charlton@unsw.edu.au (A.J.C.); g.leslie@unsw.edu.au (G.L.); 2 Eurecat, Centre Tecnològic de Catalunya, Water, Air and Soil Unit, 08005 Manresa, Spain; gaetan.blandin@eurocat.org

**Keywords:** forward osmosis (FO), computational fluid dynamics (CFD), spacer design, draw channel contraction, pressure assisted osmosis, concentration polarisation (CP)

## Abstract

Forward osmosis (FO) modules currently suffer from performance efficiency limitations due to concentration polarisation (CP), as well as pressure drops during operation. There are incentives to further reduce CP effects, as well as optimise spacer design for pressure drop improvements and mechanical support. In this study, the effects of applying transmembrane pressure (TMP) on FO membrane deformation and the subsequent impact on module performance was investigated by comparing experimental data to 3D computational fluid dynamics (CFD) simulations for three commercial FO modules. At a TMP of 1.5 bar the occlusion of the draw-channel induced by longitudinal pressure hydraulic drop was comparable for the Toray (16%) and HTI modules (12%); however, the hydraulic perimeter of the Profiera module was reduced by 46%. CFD simulation of the occluded channels indicated that a change in hydraulic perimeter due to a 62% increase in shear strain resulted in a 31% increase in the Reynolds number. This reduction in channel dimensions enhanced osmotic efficiency by reducing CP via improved draw-channel hydrodynamics, which significantly disrupted the external concentration polarization (ECP) layer. Furthermore, simulations indicated that the Reynolds number experienced only modest increases with applied TMP and that shear strain at the membrane surface was found to be the most important factor when predicting flux performance enhancement, which varied between the different modules. This work suggests that a numerical approach to assess the effects of draw-spacers on pressure drop and CP can optimize and reduce investment in the design and validation of FO module designs.

## 1. Introduction

An increasing global demand for potable water, as well as the efficient treatment of wastewater, has driven research into forward osmosis (FO) as an alternative to current processes [1]. FO provides benefits when compared to other pressure-only driven processes, such as lower hydraulic pressure and lower fouling operation, when compared to other pressure-only driven processes [2]. FO processes have been investigated widely across a range of applications and modes of operation, with pressure-assisted osmosis (PAO) and FO-reverse osmosis (FO-RO) hybrids evaluated in potential options [3,4,5,6,7]. However, concentration polarisation (CP), reverse solute diffusion (RSD) and pressure considerations within the module currently hinder greater industrial interest in the process [1,7]. 

CP is a phenomenon affecting membrane processes whereby a solute gradient forms close to the surface of, or within a membrane that hinders flux performance. External concentration polarisation (ECP) is found in all membrane processes and is a buildup of solutes within the boundary layer of the membrane surface, which lowers flux performance and hinders the concentration gradient that drives FO processes. Internal concentration polarisation (ICP) is a concentration gradient within the membrane support layer that is specific to FO processes. The ICP gradient occurs within the support structure of the membrane which, in turn, acts against the osmotic gradient which drives FO processes [1,8,9]. ICP is an inherent problem with the structure of the membrane and is reported to be unmitigated with an optimisation of operating conditions alone [8]. Additionally, ICP is reported as the main CP factor impacting flux performance in FO processes when compared to ECP, especially when the membrane active-layer faces the feed-side [10,11]. To assess the impact of CP on FO processes, numerical models have been developed to account for ICP and ECP’s effect on flux [8,10]. These CP models were developed by a modulus approach, whereby depending on the membrane orientation, a modulus was determined for ICP and ECP (either dilutive or concentrative) [6,8,10,11,12]. The CP moduli are typically considered in pairs and during module operation in the active-layer feed side (AL-FS) mode, a combination of concentrative ECP on the feed-side and dilutive ICP on the draw-side (and vice-versa) is assumed. Novel flux models that account for wide-ranging temperatures or the diffusivity of draw solutes have since been developed to predict performance for a wider range of operating conditions [11,12]. Additionally, novel models that consider ECP on both the draw-side and feed-side of FO membranes have been developed. However, the channel hydrodynamics must be accurately known and this dual layer ECP approach has only been considered under perfectly rectangular channel conditions, only accounting for spacer effects on mass transfer with considerable simplification [13,14,15]. The current approaches assume a Reynolds number based on the overall channel flow, set this value and subsequently compare hydrodynamic outputs such as pressure and velocity profiles [15,16]. The use of CFD analysis to assist in the calculation of a Reynolds number in a spacer-filled channel has not yet been applied to the calculation of mass transfer coefficients in FO CP modelling. 

The pressure considerations required to circulate draw and feed solutions within FO (and PAO) processes are an emerging area of research. While PAO is the use of FO with applied pressure assisting flux performance, even within regular FO processes it has been observed that 0.5 bar of pressure is needed to circulate fluid in 8-inch FO modules [17]. The resultant transmembrane pressure (TMP) has been found to cause the deformation of the membrane into the draw-channel, resulting in the draw-channel contraction recently studied in the literature [18,19,20,21]. Furthermore, the applied TMP has been observed to cause a long-term compaction of the membrane after operation with moderate (1–2.5 bar) pressure [3]. The transport properties of FO membranes under applied pressure have recently been explored, finding no significant changes until the active layer is compromised [22]. However, the effects of mechanical strain on flow profiles and CP have not been well established in the literature. Membrane processes usually require a spacer to provide mechanical support and the separation between membrane channels in a module [23]. This has led to spacer designs that offer low mechanical support as a tradeoff for unimpeded fluid flow, and hence a lower pressure drop [17,18,21]. While the impact of draw-spacer contraction has been reported on pressure drops and fouling potential, the effects studied have been on the relative contribution of deformation against CP effects [19].

CFD has been utilised as a means of exploring detailed hydrodynamic assessments in FO processes [18,24]. CFD can provide information about membrane modules that is not experimentally available, such a turbulence analysis through Reynolds number distributions, velocity profiles as well as shear strain analysis [25,26]. Shear strain has a known relationship to fouling and ECP mitigation in membrane processes, leading to its use in the literature to inform spacer and module design predictions [27,28,29]. Additionally, shear strain has been reported to significantly impact flux improvements in FO processes [30]. However, further knowledge of the limitations of the relative importance of Reynolds number and shear strain analysis is still needed to further optimise how effectively CFD analysis can enhance spacer design.

This paper aims to firstly assess the degree of deformation under applied TMP within commercially available FO membrane modules. Secondly, this paper will provide a detailed hydrodynamic assessment of the effects of TMP on the draw-channel of commercial FO modules using CFD. Lastly, this paper aims to numerically assess the efficiency and CP in the modules and subsequent effects under applied TMP, hence establishing a quantified relationship between TMP and CP. The link between a CFD assessment, deformation and CP aims to provide a means by which improvements can be predicted and assessed without the need for the costly production and experimental testing of the spacer designs across FO modules.

## 2. Materials and Methods 

### 2.1. CFD Modelling of Membrane Processes 

We developed 3D CFD models for 3 commercially available FO modules, 2 SW modules (a CTA from HTI (Albany, OR, USA) and TFC from Toray (Toray Chemical Korea Inc., Seoul, Korea) as well as a PF module from Porifera (San Leandro, CA, USA)). CFD modelling of three FO module processes was based on the method initially developed in [21], with modifications for further geometry and design parameters of the draw-channel. All 3D CFD simulations were performed using ®Ansys Fluent v19.1 (Canonsburg, PA, USA). CFD simulations were validated against data from previous studies, with a summary of the main operating conditions used in Table 1 [17,18,21]. 

**Figure 1:**
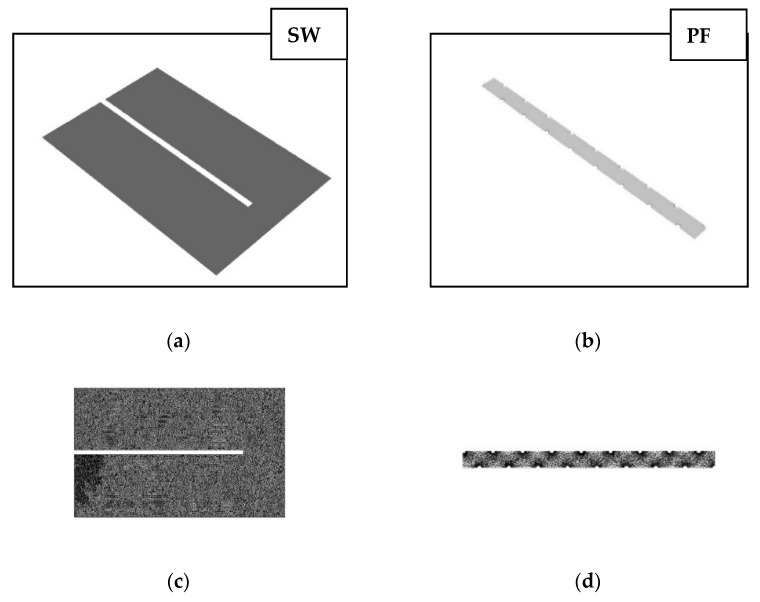
The 3D domains for computational fluid dynamics (CFD) geometry of forward osmosis (FO) modules (**a**) spiral-wound (SW) geometry (**b**) plate-and-frame (PF) geometry (**c**) SW mesh (**d**) PF mesh.

Tetramesh was employed for each fluid domain, with an average size of 0.2 mm as well as first layer inflation of 0.1 mm. In line with previous methodology, a mesh independence test was conducted by varying the mesh size from 0.1–0.5 mm with no significant variation in pressure drop observed [18,21]. The 3D domain of the spiral-wound (SW) draw-channel has dimensions of 1 m × 1.5 m for the 8 inch modules, assumed from membrane length, glue line and total area data published previously [17,21] (shown in Figure 1a, with the mesh outlined in Figure 1c). The membrane displacement of the SW channel was determined as a total height change, under the assumption of an average displacement given the fine mesh spacer [18]. The porosity change under membrane displacement of the SW channel was calculated using the following equation:

(1) $$\varepsilon^{\prime }=1-\frac{h_{p}\left(1-\varepsilon \right)}{h_{p}-\Delta h}$$

where ε is the porosity, $h_{p}\;$ is the height of the permeate channel and $\varepsilon^{\prime }$ is the adjusted porosity. However, owing to file size limitations in Ansys, only a portion of the plate-and-frame (PF) draw-channel was represented as a segment of the total channel (120 mm × 40 mm) for the PF module (Figure 1b) with inflation layers of the mesh around the spacers shown in Figure 1d. A no-slip wall condition was utilised to estimate the continuous flow between fluid and spacer, and the two sides of the membrane will be considered symmetrical for the purpose of simplification. Simulations were run using the Semi-Implicit Method for Pressure Linked Equations (SIMPLE) algorithm for pressure-velocity coupling and First Order Upwind (FOU) algorithm for the discretisation of the conservation equations with a convergence criteria of below $10^{-4}\;$ residual error term limit [18]. The PF draw channel was simulated in ANSYS as a single segment, as the flow profile does not change across the module with respect to width, and pressure drop is linear [21]. Simulations were run on a CPU-cluster with 4 Intel Xeon cores and 32 GB of ram utilized. 

### 2.2. CP Analysis through Efficiency and Modulus Characterisation

The experimental data presented in this study are extracted from previous studies, whereby the membrane active-layer faced the feed-side (for desalination and high fouling solutions) [3,17,21]. Additionally, most of the CP models in the literature typically agree that dilutive ICP occurs within the support structure of the membrane on the draw-side, and ECP occurs in the boundary layer on the feed-side [6,10,11,12,19]. As the feed solution used is deionised water the, ECP in the feed can be neglected (i.e., no solute in the feed). The ECP in the draw is characterised using a modulus approach from the literature that applied the same principles as the feed-side modulus [13]. This paper uses the CP modulus as presented in most CP studies. All experiments were performed at room temperature and with Red Sea-salt (RSS) (35.5 g/L). Current literature models explore ECP using draw channel hydrodynamics such as Reynolds number, which assume simple channel geometry (ignoring membrane deformation) and the effects of spacers. The ECP Reynolds number in this study was calculated using the CFD and deformation data presented in Section 3.1, Section 3.2 and Section 3.3 unless otherwise stated to provide a novel CFD-assisted CP model. While the full CP calculation methods can be found in the literature, a summary of the exact equations used is given below.

Starting with the basic equation for flux in an osmotically driven process, where the effects of CP are ignored and only osmotic and hydraulic pressures are taken into consideration, as shown in Equation (2).

(2) $$J_{w}=A\left(\pi_{D,b}-\pi_{F,b}+P_{feed}-P_{draw}\right)$$

where $P_{feed}$ and $P_{draw}$ are the hydraulic pressures of the feed and draw, respectively, $J_{w}$ is flux, *A* is the pure water permeability coefficient, $\pi_{D,b}\;$ is the osmotic pressure of the draw solution and $\pi_{F,b}$ is the osmotic pressure of the feed solution. 

The overall flux efficiency (*E*) was determined as a measure of the flux when compared to the maximum achievable under a specific hydraulic and osmostic driving force. The OE was determined to characterize an overall efficiency baseline of a membrane process and can be calculated as the experimental flux obtained, as a percentage of the maximum achievable flux from Equation (3).

(3) $$E=\;\frac{J_{w,\;experimental\;}}{A\left(\pi_{D,b}-\pi_{F,b}+P_{feed}-P_{draw}\right)}$$

The osmotic efficiency (*OE*) was determined as a measure of the effectiveness of the osmotic component of the driving force in Equation (2), and is determined by assuming the hydraulic driving force is negligibly losing efficiency to CP in a deionized water feed solution (i.e., no feed-side solute). The osmotic efficiency is extracted from Equation (3), and is shown in Equation (4).

(4) $$OE=\;\frac{J_{w,\;experimental\;}}{A\left(\pi_{D,b}-\pi_{F,b}\right)}-\;\frac{\left(P_{feed}-P_{draw}\right)}{\left(\pi_{D,b}-\pi_{F,b}\right)}$$

The solute resistivity to the membrane, “*K*”, which is a measure of ICP in the support layer to be used in the final flux equation. *K* is calculated experimentally, shown in Equation (5):

(5) $$K=\frac{S}{D}=\;\frac{t\tau }{D\varepsilon }$$

where S is the structural parameter of the membrane, D is the solute diffusion coefficient, t is the tortuosity, ε  is the thickness, and τ is the porosity. The *S* parameters used in this study were drawn from the literature and assumed to remain constant.

The experimental determination of *K* is assessed using the experimental flux, fitted to Equation (6).

(6) $$K=\frac{1}{J_{w}}\ln\left(\frac{A\pi_{D,b}+B}{B+J_{w}+\;A\pi_{F,b}\;}\right)$$

where B is the solute permeability, and *K* is determined as an average across the experimental range tested. 

The ICP modulus can then be calculated as a ratio of the osmotic pressure gradient within the membrane itself, shown in Equation (7).

(7) $$\frac{\pi_{D,m,i}}{\pi_{D,m}}=\;e^{-J_{w}K}$$

where $\;\pi_{m,i}$ is the osmotic pressure of the support layer against the active layer, $\pi_{D,m}$ is the actual osmotic pressure on the membrane surface.

The effects of ECP are first characterised using the mass transfer coefficient k, based on the CFD hydrodynamic outputs as the channel shape is irregular shown in Equation (8).

(8) $$k\;=\frac{ShD}{d_{h}}$$

where Sh is the Sherwood number, D is the solute diffusion coefficient and $d_{h}$ is the hydraulic diameter. It should be noted the Sherwood number is calculated from the fitting parameters for FO [11]. The ECP modulus for the draw-side is then calculated as shown in Equation (9):

(9) $$\frac{\pi_{D,m}}{\pi_{D,b\;}}=\;e^{-J_{w}/k}$$

where $e^{\frac{-J_{w}}{k}}$ is the ECP modulus on the draw-side. 

### 2.3. CFD Hydrodynamic Parameter Analysis 

CFD analysis provides a detailed analysis including shear strain and Reynolds number, each known to impact membrane performance. The shear strain rate exerted by the fluid on the draw-channel is a measure of the perpendicular force acting on the channel and membrane surface. Shear strain rate is a typically reported CFD output of membrane process modelling as it provides the strain rate of membrane processes with a high degree of accuracy [24,31]. Wall shear rate increases are associated with improved CP and fouling mitigation. ANSYS Fluentv.19.1 was used to generate shear rate contours of the membrane surface in the draw channels of FO modules, as well as generate average membrane surface and bulk values. This analysis was used to link CFD hydrodynamic parameters with channel occlusion and CP. Shear rate was calculated based on the method presented in the previous literature [18]; however, a summary of the method is given in Equation (10).

(10) $$Shear\;Rate\;=\;{\left[2\;\frac{\delta U_{i}}{\delta X_{i}}\right]}^{\frac{1}{2}}$$

where $U_{i}$ is velocity and $X_{i}$ is the spatial coordinate. 

Reynolds number was characterised to assess the degree of turbulence and mixing in the channel and has been reported in the CFD assessment in narrow spacer filled channels. Reynolds number is used as a channel average in CP models [10] due to links with improved CP and fouling performance in membrane processes [32]. Reynolds number was calculated as both a contour map on the membrane surface as well as average values for the bulk fluid and membrane surface. As with the shear rate analysis, Reynolds number was assessed to establish a relationship between TMP and CP through a hydrodynamic CFD analysis. The method is based on the previous literature [18]; however, a summary of the factors is given in Equation (11).

(11) $$Re=\frac{{\rho v}_{c}d_{c}}{u}$$

where ρ  is the fluid density, d  is mesh cell volume^1/3, $v_{c}$ is velocity magnitude in the cell and u is the fluid viscosity. Note the difference between a CFD cell-volume-based value and the value given in ECP models, based on hydraulic diameter (Equation (12)).

(12) $$\;Re_{d}=\frac{\rho_{d}v_{d}d_{h}}{u_{d}}$$

where $\;Re_{d}$ is an overall Reynolds number for the entire draw channel, $d_{h}$ is the hydraulic diameter, typically expressed as a rectangle in FO processes ($\frac{2WH}{W+H})$ and the rest of the parameters are all expressed as channel averages (where W and H are the width and height of the channel, respectively).

## 3. Results and Discussion

### 3.1. CFD Model Validation and Membrane Deformation Analysis 

TMP in FO processes has been shown to cause draw-channel occlusion, whereby the membrane deforms into the draw channel [18,19,21]. To determine the exact level of occlusion experienced in a range of FO modules under pressure, models were developed using experimental data at 0 bar and 1.5 bar of TMP. Firstly, the draw-channels were modelled as 3D CFD geometries and validated against a range of experimental data reported previously in the literature to assess the degree of membrane deformation across module and membrane types [17,21]. The PF (Porifera) module was validated from previous experimental data, using the method from our previous work [18]. However, a novel 3D geometry for a channel occlusion of 49% was developed to determine the membrane deformation at 1.5 bar of TMP, to establish a comparison point between the three commercially available FO modules. 

**Figure 2:**
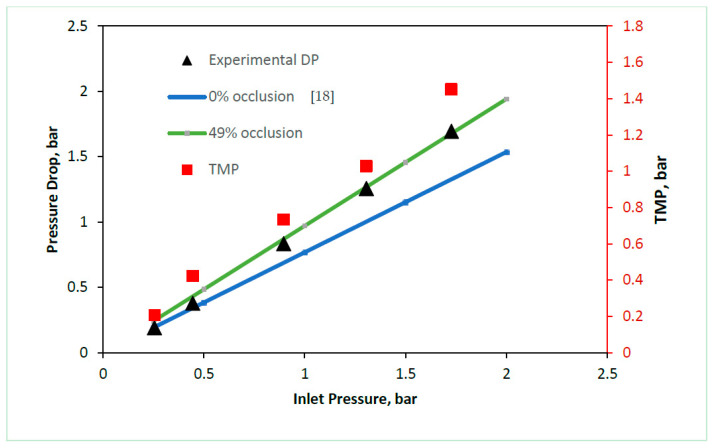
Porifera PF draw-channel CFD validation of draw channel pressure drop against draw inlet pressure, showing draw channel contraction, validated against experimental data previously reported [18].

The Porifera module (Figure 2) shows pressure-drop behavior following the 0% channel at lower TMPs (between 0–0.5 bar), and with increasing pressure, the pressure drops shift to match the behavior of the 49% channel, between TMPs of 0.8–1.5 bar. The 49% channel occlusion is high when compared to the relatively low TMP applied, and is due to the lack of mechanical support from the dot-spacer type used in the module.

The SW modules were simulated as 3D geometries of a single sheet, using a pressure-drop comparison method previously established in the literature [17]. The SW modules were both modelled from a different dataset to the PF module, where the only the TMP was varied (and not the inlet pressure). This resulted in the “calibration” approach whereby after the 3D dimensions were established, the open channel porosity was calibrated, the pressure was dropped at 0 bar TMP and the channel height was varied until the pressure drop matched at 1.5 bar TMP.

**Figure 3:**
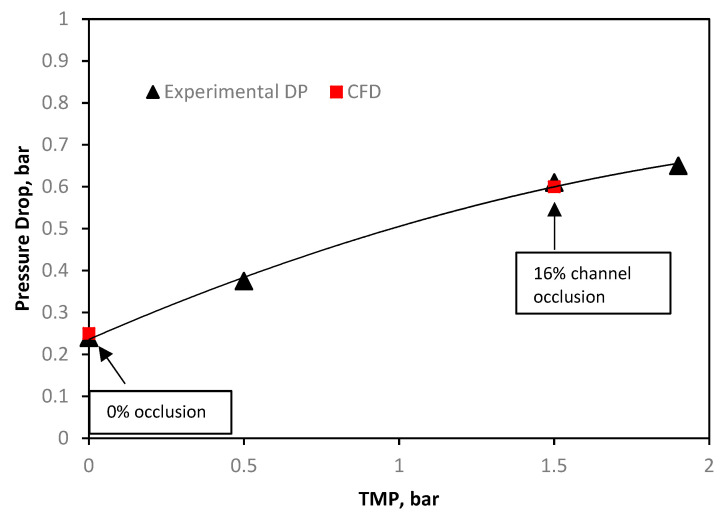
Toray SW draw-channel computational fluid dynamics (CFD) validation of the draw channel pressure drop against inlet pressure, showing draw channel contraction, validated against experimental data previously reported [17].

The Toray 0% occlusion channel (Figure 3) was calibrated against the experimental channel at negligible TMP. The initial pressure drop is caused by the resistance of the spacer in the draw-channel. At 1.5 bar of TMP, the channel height was contracted such that the experimental pressure drop matched a channel that was occluded at 16%. The lower degree of occlusion shown in the Toray module when compared to the Porifera module (Figure 2) is due to the increase mechanical support of a fine mesh draw-spacer.

**Figure 4:**
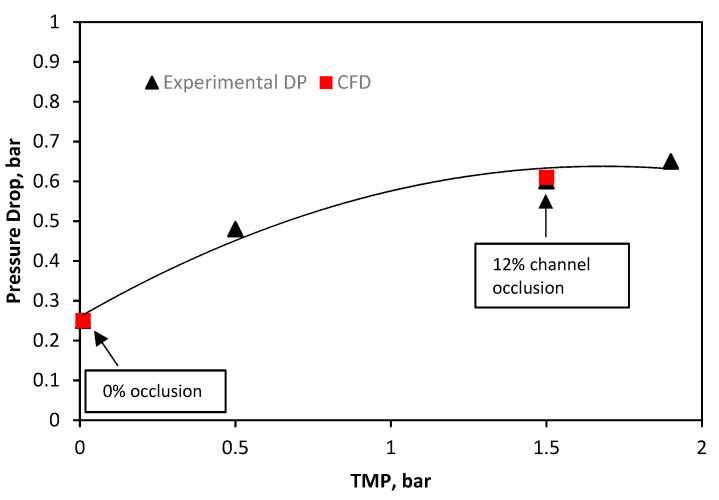
HTI SW draw-channel CFD validation of draw channel pressure drop against inlet pressure, showing draw channel contraction validated against experimental data previously reported [17].

The CFD validation of the HTI module (Figure 4) was from the same dataset as the Toray module, and as such follows the same format. The CFD open channel (0%) was calibrated against the experimental data at negligible TMP and demonstrates a similar pressure drop to the other SW module (Toray, Figure 3). At 1.5 bar of TMP, the experimental pressure drop matches a CFD geometry of 12% occlusion. The HTI spacer is a dense/rigid Tricot spacer, with the highest degree of mechanical support provided across the three modules [17]. The mechanical support of the dense HTI draw spacer allows for the least deformation, and therefore least channel occlusion (12% at 1.5 bar TMP). Furthermore, the Porifera “dot-spacer” design for the draw-channel offers by contrast the least mechanical support [18], and subsequently the highest (49%) channel occlusion under pressure. Overall, the three FO modules clearly demonstrate a region of deformation, whereby the pressure-drop change with inlet pressure is high, physically representing the membrane moving closer to the draw-channel spacer for mechanical support. Subsequently (but not shown within the reported TMP range in this study), the pressure-drop gradient is lower; while some further membrane deformation may occur, the increase in occlusion at higher TMP is significantly slower and expected to reach insignificance since the membrane rests fully against the spacer or channel wall. From these results, a comparison point at 1.5 bar TMP is now used for the rest of the paper to investigate the effects of TMP on hydrodynamics, CP, and therefore overall flux performance.

### 3.2. Shear Strain Analysis 

Utilising the two comparison points of channel/membrane behaviour at 0 and 1.5 bar of TMP, the effects of TMP on draw channel hydrodynamics can be assessed in detail by 3D CFD simulations. Shear strain analysis is an assessment of the force exerted by a fluid on the membrane surface, previously reported in the literature for its effects on fouling and more importantly, ECP [18,27,28]. The effects of TMP on the shear rate at the membrane surface, as well as the bulk fluid, are further investigated and linked with CP considerations directly in later sections of this study.

**Figure 5:**
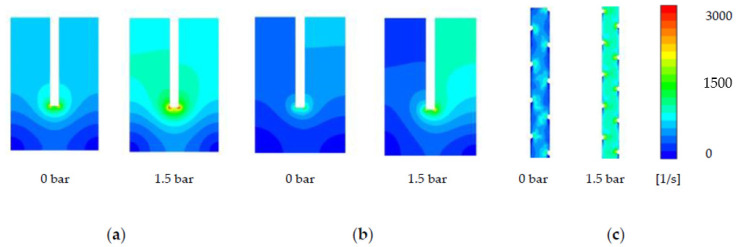
Effect of transmembrane pressure (TMP) increase on shear strain rate at the membrane surface, with the right and left simulations 1 and 1.5 bar TMP respectively for (**a**) Toray (**b**) HTI (**c**) Porifera.

The Toray module (Figure 5a) shows the most significant change of the SW modules to shear rate on the membrane surface under the effects of 1.5 bar TMP. The membrane shear strain rate distribution at 0% occlusion shows high shear stress (657 ± 63 $s^{-1}$) within the straight channel of the membrane leaves, and dead-zones of lower shear rate as expected in the corners (13 ± 25 $s^{-1}$). At 16% (1.5 bar TMP) occlusion, the straight channels that cover the majority of the shear distribution demonstrate a shear rate increase to 825 ± 63 $s^{-1}$, as expected of a narrower channel where the fluid local cross flow velocity (CFV) increases in proportion to a lower cross-sectional area. The HTI demonstrates a more uneven distribution of shear strain on the membrane surface (Figure 5c), and a lower initial shear stress when compared to the Toray module (375 ± 63 $\;s^{-1}$). This uneven shear strain distribution is likely due to the denser spacer, creating more flow resistance and higher gradients of strain (noting that all simulations were run at 0.1 m/s). The shear strain increase with a 1.5 bar TMP of an applied to the HTI membrane increases the majority of the shear stress in the straight channels to (657 ± 63 $s^{-1}$). The Porifera membrane has the least consistent shear stress distribution on the membrane surface, with each spacer creating a shear “dead-zone” along the length of the channel (Figure 5c). This inconsistency leads to two visually dominant shear distributions of 657 and 825 ± 63 $\;s^{-1}$ within the 0% channel. The Porifera module has the least dense spacer of the three modules, and the low mechanical support creates a wider distribution and a much higher number of dead-zones. All three modules, however, overall demonstrate significant shear increases on the membrane surface with applied TMP pressure of 1.5 bar.

**Figure 6:**
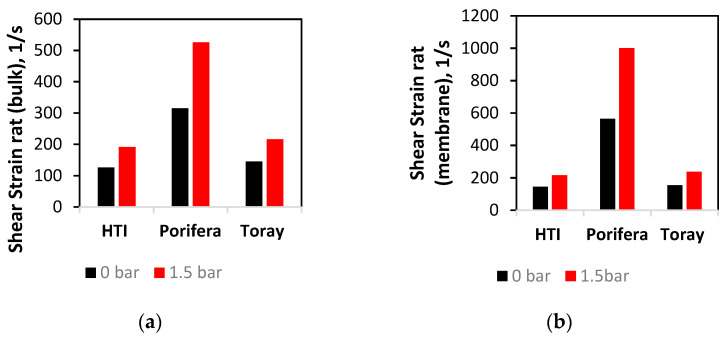
Effect of TMP increase on shear strain at the (**a**) bulk fluid flow and (**b**) membrane surface at 0 and 1.5 bar of applied TMP.

Figure 6 shows the average values of the shear rate in the bulk average of the fluid, and represents the average perpendicular force exerted fluid in the draw-channel. The Porifera module is observed with the highest average shear rate in both the bulk fluid and at the membrane surface (315 and 526 $\;s^{-1}$), matching the observed trend on the membrane surface (Figure 5). The Toray has the lowest shear values, further matching the open nature of a larger channel. Overall, the three modules experience an increase in shear rate at both the bulk flow and membrane surface when TMP is applied. Additionally, the shear rate is much higher at the membrane surface, even with a relatively low CFV of 0.1 m/s and narrow channel size (1–3 mm across the modules). Additionally, on both the membrane and bulk fluid, an applied TMP of 1.5 bar increases the shear by more than double for all the modules studied. This shear increase has implications on efficiency and CP performance, to be further investigated in this study.

### 3.3. Reynolds Number Analysis of Flow 

ANSYS Fluent was used to calculate the Reynolds number distribution on the membrane surface, as well as the membrane average and bulk average values to produce a detailed assessment of turbulence in the modules, under the effects of applied TMP (1.5 bar). The assessment of Reynolds number in the draw-channel aims to provide a complimentary assessment to shear strain and further detail to an overall hydrodynamic analysis.

**Figure 7:**
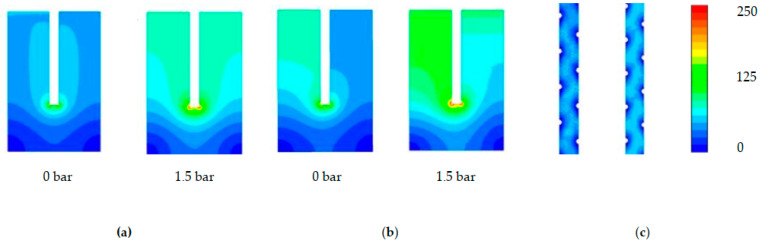
Effect of TMP on Reynolds number on the membrane surface with the right and left simulations 1 and 1.5 bar TMP, respectively, for (**a**) Toray (**b**) HTI (**c**) Toray.

The Toray shows the lower Reynolds distribution (Figure 7a) of the SW modules, with the straight channels of the membrane’s leaves showing symmetrical and majority values of 56 ± 13. Symmetrical dead-zones of low turbulence are found in the corners of the membrane sleeves, as well as a slight increase to 81 ± 13 on the corner of the glue-line, with an observed increase in the severity of the glue-line turbulence between the 0% and 12% channels. An increase of Reynolds number to 69 ± 10 occurs under a TMP increase of 1.5 bar, an explanation of which is the increase in fluid velocity due to a decrease in the cross-sectional area (similar to Section 3.2). The HTI module has the most extensive range in the distribution of the Reynolds number across the membrane surface (Figure 7b). Furthermore, the range across the outlet channel of the membrane leaf is of a much higher range than the Toray module (10 vs 68 ± 10). This phenomenon can be explained by the less dense spacer of the Toray draw module, and hence emphasises the importance of contour maps as a means of measuring Reynolds distribution in FO modules with larger draw-channels (Section 3.1) [17]. 

**Figure 8:**
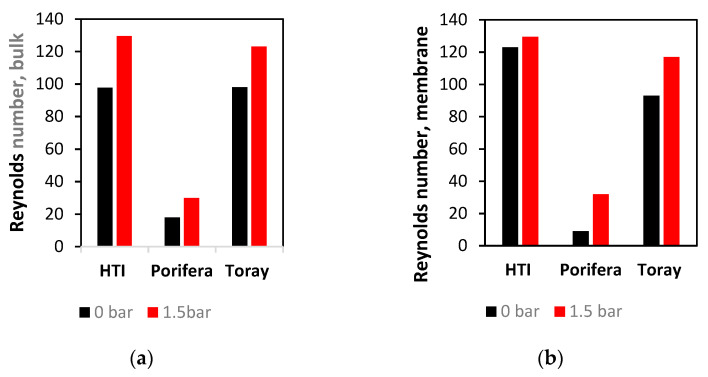
Effect of TMP on Reynolds number in the (**a**) bulk fluid flow (**b**) membrane surface compared at 0 and 1.5 bar of applied TMP.

In direct contrast to the shear data (Section 3.2), the Porifera module has the lowest average Reynolds number at any TMP in the bulk fluid flow (Figure 8a). Additionally, at the membrane surface, the Porifera module is observed as the lowest Reynolds number, and when TMP of 1.5 bar is applied (Figure 8b), it also shows the least significant increase under applied pressure. Hence, the Porifera module Reynolds number is the least sensitive to deformation, likely due to the low degree of mechanical support offered by the draw-spacer and lack of turbulence promoters evenly distributed like a SW mesh spacer. Additionally, the turbulence is much lower on the membrane surface, an opposing trend to the shear strain data (Section 3.2). Overall, the detail shown in Figure 7 presents a similar contour profile to the shear strain data shown in Figure 5 but with less severity in the effect of TMP (with the exception of the Porifera module). This detailed assessment of turbulence through Reynolds number will be further characterised and linked to CP later in this study.

### 3.4. TMP Effects on CP 

The impact of TMP on deformation and subsequent draw channel contraction clearly affects the hydrodynamics within FO modules; as such, an assessment of applying TMP on overall efficiency and CP is proposed. The CP effects are then linked to the CFD hydrodynamic analysis from Section 3.2 and Section 3.3 later in this study. 

Firstly, the PF and SW flux data were assessed by expressing the flux as a percentage of the maximal possible flux to determine the overall flux efficiency (Equation (3)) from experimental data previously reported in the literature [3,17,21]. The flux data were then used to determine osmotic efficiency, which characterizes the effectiveness of the osmotic component in the driving force (Equation (4)). Subsequently, the flux data were processed in a CP modulus model [10] to characterise both ICP and ECP. The flux and osmotic efficiency is then compared against ICP and ECP across a range of TMPs, expressed as a percentage change from initial conditions to normalise different operating conditions.

**Figure 9:**
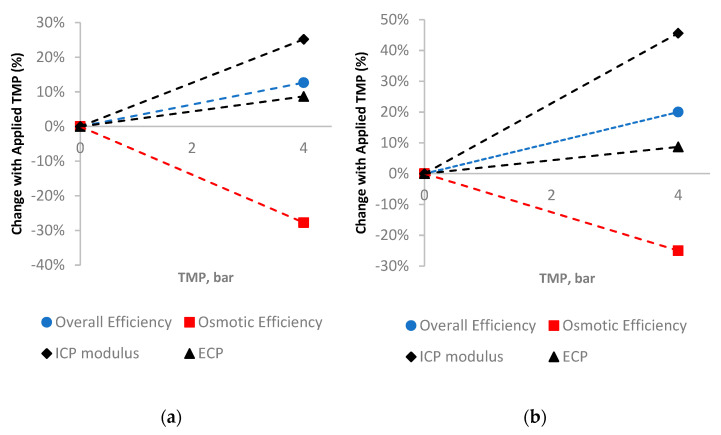
An assessment of efficiency and CP (ICP and ECP) for membranes out of a module and in a cross-flow cell (**a**) HTI (**b**) Porifera modules. Values are expressed as percentage changes from initial conditions at 0 bar TMP, to normalize initial membrane conditions and characteristics.

**Figure 10:**
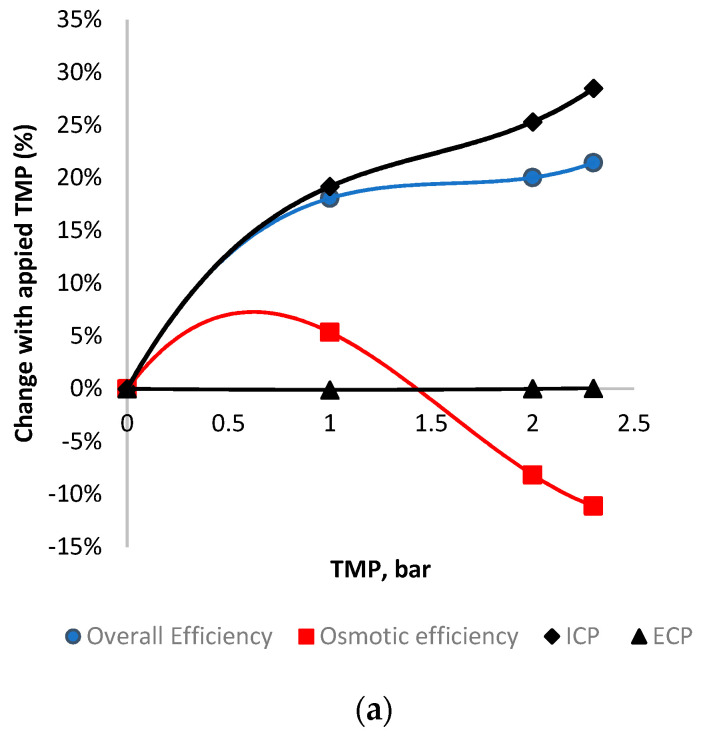

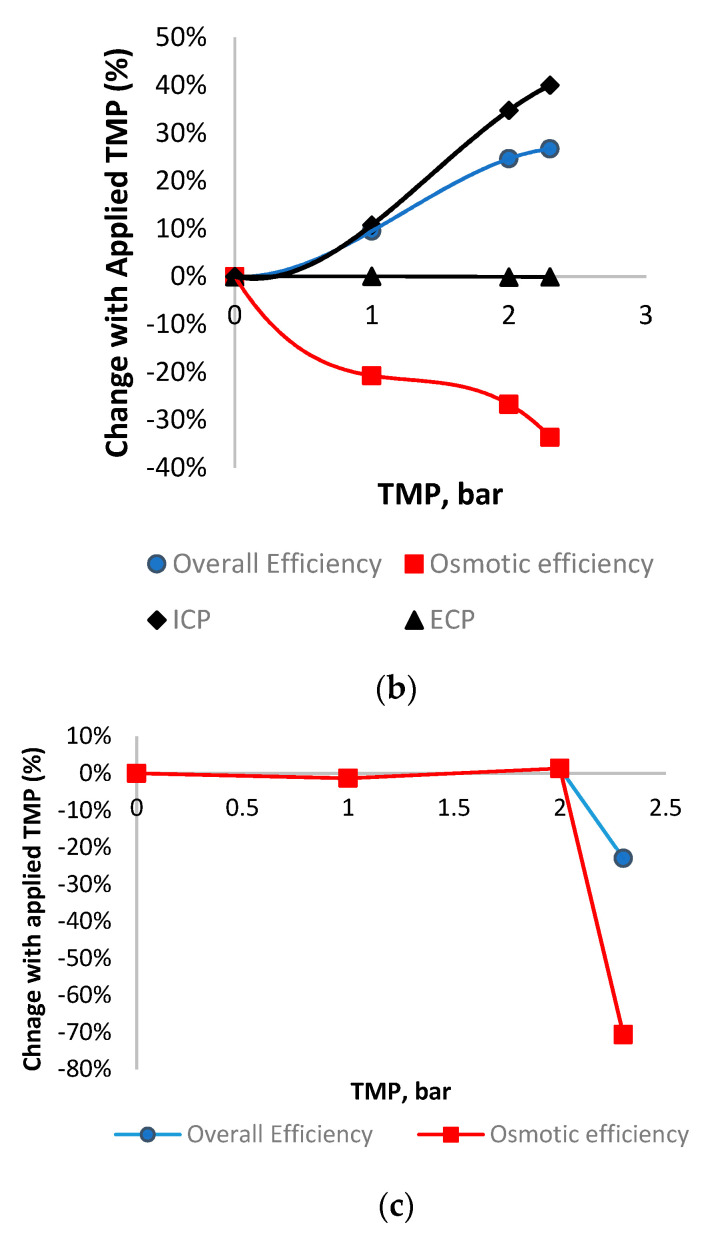
An assessment of the change in efficiency and CP (ICP and ECP) with applied TMP for module scale (**a**) HTI (**b**) Toray (**c**) Porifera modules expressed as percentage changes from initial conditions at 0 bar TMP.

The efficiency and CP analysis was first assessed on the membrane scale, where data from the literature were used in this study, given at 0 and 4 bar and studied in a small-scale crossflow cell [3] (Figure 9). The data are expressed as a percentage change from conditions at 0 bar TMP (FO mode) to normalize the initial operating/membrane characteristics and allow for a direct comparison between membrane and module scale. Figure 9a is the HTI membrane and illustrates the overall trends to be expected for FO membranes operated under TMP. The overall efficiency increases by 13%, with the addition of hydraulic pressure in an FO process (whereby the hydraulic component of the driving force is almost 100% efficient). The osmotic component of the driving force decreases as the higher pressure causes higher flux, and, thus, the osmotic pressure is hindered by CP. This increase is illustrated in Figure 10a in both the ICP and ECP increase of 25 and 8%, respectively, as expected with increasing flux [10]. The Porifera membrane has a higher hydraulic permeability [3], and so higher flux with TMP; thus, the CP effects are greater with higher pressure/flux. The Porifera membrane has a 46% increase in ICP, yet this is balanced with a higher overall flux efficiency increase, as the effects of CP are balanced against the hydraulic permeability. 

The efficiency and CP analysis was then performed at the module scale, to determine the effects the different module and spacer designs exhibit under applied TMP, shown in Figure 10. The analysis was again performed on flux data from the literature, expressed as percentage changes from initial conditions at 0 bar to allow for direct comparison. 

Figure 10a illustrates the efficiency and CP effects on a module scale for the HTI module, calculated based on the flux data from the literature [17]. The TMP of 0–2.3 bar is a narrower range than the membrane-scale shown in Figure 10a, but suits the purpose of analyzing the efficiency and CP during the membrane deformation and compares well to the overall picture given over a larger range of TMP’s. The overall flux efficiency increase found by adding hydraulic pressure into the HTI SW module was over 20% from 0–2.3 bar, matching the trends of the membrane-scale results. However, it is clearly observed that much of the efficiency gain is from 0–1 bar of TMP, with lower subsequent increases (less than 2% improvement over the subsequent 1 bar of applied TMP). Unexpectedly, the osmotic efficiency of the HTI module increases within the 1 bar TMP range, in contrast to the trends predicted from the membrane-scale analysis (Figure 9). The osmotic efficiency then decreases rapidly from 5% to -8% over the next 1 bar of TMP. An explanation for the unexpected increase in osmotic efficiency can be found by observing the TMP band at which the improved performance occurs, noting that it matches with the deformation region of the HTI membrane (Section 3.3). This unexpected trend indicates that the deformation has a positive effect on osmotic and overall flux efficiency. With respect to the CP analysis, the ICP increases proportionally with increased TMP (and, thus, flux), as expected given the relatively constant nature of the S parameter in FO membranes [10,17]. In contrast to the ICP, the ECP remains constant across the TMP’s, and does not increase as expected with higher TMP/flux. This stable ECP is in direct contrast with the increasing ECP effects under applied TMP in the membrane scale (Figure 9). An explanation for this is the changing hydrodynamics in the draw channel, whereby TMP causes contraction, a narrower channel and a faster CFV. This fast CFV and decreasing CFV mean that the changing hydrodynamics outweigh the flux increase effects on ECP. Specifically, the ECP is held constant by the higher Reynolds number, and, as such, the higher “*k*” value counterbalancing the increase usually expected by higher flux. The Toray module demonstrates the same increase in overall efficiency within TMP’s of 0–2.3 bar; however, with a much slower increase than the HTI at pressures of 0–1 bar (Figure 10). This is coupled with an osmotic efficiency that decreases, yet the decrease slows at TMPs of 1–2 bar of TMP, indicating an optimum point of operation. The Toray module displays similar behavior to the HTI overall, with an optimum region of higher-than-expected performance matching with the deformation region of the membrane. However, the optimum range of the Toray module is found at larger TMPs than the HTI due to less dense spacer support (Section 3.1). ECP decreases only slightly with applied TMP, as with the HTI module. The Porifera module (Figure 10c) has no CP data points illustrated, as the much higher deformation and resultant channel occlusion were the primary factor for the flux and this efficiency loss. This is particularly apparent as the overall efficiency does not increase with applied TMP, in direct contrast to the Toray and HTI modules. The efficiency data is also in direct contrast the Porifera membrane-scale data (Figure 9), indicating that the occlusion is therefore not spread evenly and is likely contacting the spacer or wall results in membrane area (and, hence, the flux performance loss). It is important to note the low TMP pressures recommended for Porifera operation [3]; however, this indicates the importance of mechanical support in the FO modules when the PAO mode is used. 

This first implication of the data overall is the importance of ECP when considering FO membranes and modules, illustrating how channel hydrodynamic impact and can mitigate ECP on the draw-side. The effect of membrane deformation on channel hydrodynamics and its subsequent impact on CP is an emerging idea previously reported in the literature [19]. However, the CP models mentioned previously do not account for how membrane deformation would affect the flow profile, and do not use CFD analysis to assist in CP characterisation. The next section of this study will further assess this relation by linking the CFD hydrodynamic analysis to the unexpected CP trends under applied pressure. 

With the detailed assessment of the level of occlusion and hydrodynamic characterisation by CFD (Section 3.1, Section 3.2 and Section 3.3), the analysis of the efficiency and CP (Section 3.4) can be compared and linked. Current numerical CP models do not account for ECP based on irregular flow profiles caused by deformation, and CFD analysis can provide a link to assess CP improvements by deformation. The results are compared across the FO modules to assess the relationship between the CFD characterisation of the draw channel flow profile, to link against experimental and CP analysis to improve further the effectiveness and convenience of CFD in future module and spacer design.

The CFD, efficiency and CP analysis are summarised in Table 2. The HTI module (under 1.5 bar TMP) has an improved osmotic efficiency, in part due to the improved draw-channel hydrodynamics (Section 3.4). This is in direct correlation with the shear force and Reynolds number improvement in the module under 1.5 bar TMP (Table 2). Given the high osmotic and overall efficiency performance of the HTI module, the higher bulk Reynolds number increase over the Toray can be stated as the most important factor when considering the relatively similar shear strain values. However, the scope of this study does not include fouling effects whereby the strain rate is more likely to be a major factor.

The PF Porifera module, however, has the greatest Reynolds increase (40% in the bulk flow), due to the high degree of occlusion (Section 3.1) and high turbulence from the uneven flow profile (Section 3.3). However, the relationship between turbulence and efficiency is balanced against the membrane-area loss for the Porifera module, which would decrease the area possible for flux to occur. This trade-off implies that mechanical support must be balanced against flow improvements overall and depends on the TMP desired for each FO module. The HTI and Toray modules experienced lower increases in turbulence (Table 1), but due to the higher mechanical support maintained a high membrane area availability. The shear strain rate (Table 2) improves by 20%–40% across all modules, is also strongly linked to increased flux performance, with shear strain on the membrane surface likely to disrupt the ECP boundary layer on the draw-channel and explain the higher-than-expected osmotic efficiency. The significant increases in the Reynolds number and the shear strain (Table 2) seem to link more directly to osmotic efficiency in the deformation region of the membrane. As such, future spacer design should seek to improve the shear strain for the increased flux performance of the module. The spacer design can also aim to improve Reynolds number in the channel through turbulence promotion should RSD improvement be an aim. However, when comparing the Reynolds number analysis of FO channels, the findings of this study imply a less direct link than the mass transfer coefficient “*k*” (Equation (8)) from the CP models would suggest. The appropriateness of the mass transfer coefficient “*k*” using a hydraulic-diameter based Reynolds number is shown as inaccurate when the channel flow has most deviated from channel geometry assumptions, especially prominent during deformation. This indicates that ECP in the draw-channel plays a greater role in the draw-side than initially assumed [9,10,11]. Furthermore, models that account for ECP in the draw-channel should further aim to take into account irregular (nonrectangular or perfectly spherical) channel shapes with the assistance of CFD to determine a draw-channel flow profile. Overall, most notably, it can be seen that the overall CP effects decrease within the deformation range of the membrane modules. With 1.5 bar across all modules, the effects of the CP are lower; as such, the membrane deformation region is likely an efficient operating point of the FO modules when in FO and PAO modes. 

## 4. Conclusions

By comparing pressure loss data, this study determined the levels of occlusion of commercially available FO modules at 1.5 bar of TMP to be 16, 49 and 12% between the Toray, Porifera and Toray modules, respectively. The difference in occlusion between the modules was explained by observing the degree of mechanical support that the draw-spacers offered. Shear strain contour maps from CFD demonstrated a consistent increase in shear strain across all three modules, with an average increase of 62% at the membrane surface under 1.5 bar TMP. Reynolds number demonstrated a consistent increase across the modules, with an average increase of 31% in the draw-channel across the modules under 1.5 bar TMP. An assessment of efficiency and CP showed overall efficiency increased in the SW modules at the region of membrane deformation under TMP, but not the PF module—due to a high degree of occlusion likely detracting from the membrane area. However, osmotic efficiency did not decrease consistently as expected and deformation was found to positively increase osmotic efficiency, indicating that the optimum operating pressure for FO (PAO) lies when the membrane is deforming into the draw-channel. This study found the improved hydrodynamics from membrane deformation promoted turbulence and shear strain which disrupted the ECP boundary layer and increased flux. Additionally, the hydrodynamics obtained from CFD assessment such as the Reynolds number were incorporated into the ECP model to improve accuracy over simplified hydraulic calculations used currently. The implications for this work extend into the further improvement of draw-spacer design, where the lessons learned mean that predictions can be made from CFD, and assessed before costly and time-consuming experimental testing.

## Figures and Tables

**Table 1:** Summary of operating conditions used in CFD simulation and numerical analysis, validated against previous experiments in the literature [17,18,21].

Specification	SW’s	Porifera (PF)
Membrane	Toray-CSM FO 8-inch HTI 8 inch	PFO 20
TMP	0–2.5 bar	0–1.6 bar
CFV (Draw)	0.4–0.25 m/s	0.02–0.36 m/s
Draw spacer type	woven fiber	dot spacer
Draw solutions	35.5 g/L (RSS) Tap water	35.5 g/L (RSS) Tap water
Surface area per sheet (in module)	1.5$\;m^{2}$	1$\;m^{2}$

**Table 2:** Summary assessment of the effects of TMP on efficiency and CP of the three modules. In the draw, % calculated between 0 and 1.5 bar TMP.

Parameter	HTI	Porifera	Toray
Reynolds (bulk)	25%	40%	20%
Reynolds (membrane)	10%	72%	10%
Shear strain (bulk)	34%	41%	33%
Shear strain (membrane)	33%	44%	35%
ICP	21.9%	n/a	22.3%
ECP	0%	n/a	−2%
Overall Efficiency	19.4%	0.01%	17.8%
Osmotic Efficiency	1%	0.01%	−22.2%
RSD	−7% *	−16% *	n/a

* Data determined from experimental results previously reported [3].
